# Evaluating the antidiabetic effects of R-verapamil in type 1 and type 2 diabetes mellitus mouse models

**DOI:** 10.1371/journal.pone.0255405

**Published:** 2021-08-06

**Authors:** Yu-Syuan Chen, Shao-Ju Weng, Shu-Hsien Chang, Rou-Ying Li, Guang-Tzuu Shane, Jui-Pao Hsu, Sheng-Wen Yeh, Ai-Ching Chang, Meng-Ju Lee

**Affiliations:** 1 Center Laboratories Inc., Taipei, Taiwan, R.O.C; 2 Lumosa Therapeutics Co., Ltd., Taipei, Taiwan, R.O.C; Stellenbosch University, SOUTH AFRICA

## Abstract

The global incidence of diabetes mellitus (DM) is increasing. Types 1 and 2 DM are associated with declining β-cell function. Verapamil (50% S-verapamil and 50% R-verapamil) can treat DM by downregulating thioredoxin-interacting protein (TXNIP), which induces islet β-cell apoptosis. However, it may also induce cardiovascular side effects as S-verapamil is negatively inotropic. In contrast, R-verapamil only weakly induces adverse cardiac effects. In this study, we aimed to determine the antidiabetic efficacy and cardiovascular safety of R-verapamil. We examined R- and S-verapamil binding through *in vitro* studies. Streptozotocin-induced type 1 and *db*/*db* type 2 DM mouse models were used to assess the antidiabetic efficacy of verapamil. IL-6, blood glucose (BG), Txnip expression, and β-cells were evaluated in streptozotocin-induced diabetic mice, while body weight, BG, and serum insulin were measured in the *db*/*db* mice. In the type 1 DM study, 100 mg/kg/day R-verapamil and racemic verapamil lowered BG, downregulated Txnip expression, and reduced β-cell apoptosis. In the type 2 DM study, the optimal R-verapamil dosage was 60 mg/kg/day and it lowered BG and raised serum insulin. However, efficacy did not increase with R-verapamil dosage. R-verapamil combined with metformin/acarbose improved BG and serum insulin more effectively than metformin/acarbose alone or verapamil combined with acarbose. R-verapamil had weaker cardiovascular side effects than S-verapamil. R-verapamil was 9.0× and 3.4× less effective than S-verapamil at inhibiting atrial inotropy and ileal contractility, respectively. It was also 8.7× weaker than S-verapamil as an agonist of somatostatin receptor type 2 (SSTR2), inhibiting ileal neurogenic contraction. Hence, R-verapamil may be an optimal DM treatment as it is safe, improves glycemic control, and preserves β-cell function both as monotherapy and in combination with metformin or acarbose. R-Verapamil has potential for delaying or arresting DM progression and improving patients’ quality of life.

## Introduction

Diabetes mellitus (DM) is a common metabolic disorder characterized by hyperglycemia. About 10.5% of the population in the USA has been diagnosed with DM [1]. Type 1 diabetes mellitus (T1DM) is caused by destruction of pancreatic β-cells, whereas type 2 diabetes mellitus (T2DM) mostly the result of insulin resistance. T2DM is the more common form and accounts for ~90% of all DM cases [2]. In both T1DM and T2DM patients, progressive β-cell failure eventually occurs. In T1DM, it is typically caused by an autoimmune attack against the β-cells. In contrast, T2DM pathogenesis is comparatively more variable and culminates in different degrees of β-cell failure.

Reduced β-cell mass or number has been detected in both T1DM and T2DM [3–5]. Recent studies have shown that many T1DM patients still possess a few functioning β-cells, even years after disease onset. The prevalence and amount of endogenous insulin secretion from these cells was shown to be higher when diagnosis occurred during adulthood [6, 7]. This preservation of insulin production was reportedly associated with better glycemic control and prevention of chronic complications [8]. In T2DM, the total islet volume and β-cell numbers were ~30% lower than those non-diabetic subjects [9, 10]. β-cell failure in T2DM was further elucidated by the United Kingdom Prospective Diabetes Study (UKPDS). After an initial decrease in HbA1c, metformin- and sulfonylurea-treated T2DM patients presented with progressive deterioration in glycemic control [11, 12]. The “relentless HbA1c rise observed with sulfonylureas and metformin” was the consequence of the progressive decline in β-cell function of T2DM. Within 3–5 years, ~50% of all T2DM patients with T2DM require another drug to maintain HbA1c < 7.0% [13]. However, there are still no effective therapies against reduced β-cell mass in T1DM and T2DM.

Apoptosis is the main form of β-cell death in both T1DM and T2DM [14], and β-cell death may be induced by thioredoxin-interacting protein (TXNIP). TXNIP was first identified as the most up-regulated gene in an oligonucleotide microarray study looking at the effects of glucose on isolated human pancreatic islets [15]. As a negative regulator of thioredoxin, TXNIP exerts its proapoptotic effects on β-cells by inhibiting thioredoxin and inducing oxidative stress, which is a key element in β-cell glucotoxicity and apoptosis. Importantly, TXNIP is highly regulated by alterations in BG levels [16–18].

Oral verapamil administration in T1DM and T2DM mouse models prevents β-cell apoptosis and promotes β-cell function [19]. Once-daily oral verapamil administration in recent-onset T1DM patients safely and effectively preserved β-cell function and reduced endogenous insulin requirement [20]. Moreover, oral verapamil was associated with lower incidences of T2DM. Our investigation based on Taiwan’s National Health Insurance Research Database (NHIRD) disclosed that verapamil could significantly lower the incidence of newly diagnosed T2DM compared to other prescribed calcium channel blockers (CCBs) [21]. Foregoing studies revealed that verapamil is a potential DM prevention and treatment strategy and might be superior to other CCBs.

Verapamil is a first-generation CCB that prevents calcium ions from entering slow L-type (long-acting) calcium channels during depolarization in the vascular smooth muscle and myocardium [22]. Verapamil was approved by the U.S. FDA in 1981 for the treatment of angina, hypertension, supraventricular tachycardia, and atrial fibrillation. As DM is a chronic disease, verapamil is advantaged with the combination of DM treatment and cardiovascular (CV) disease prevention, it might be considered as a promising drug candidate for DM.

Several studies indicated that R-form verapamil (R-Vera) is efficacious and has relatively few CV side effects [23, 24]. Chiral drugs now comprise 40–50% of the market for various therapeutic classes [25]. The pharmaceutical industry has increased its focus on the development of enantiomerically pure drugs as they have clear advantages such as superior potency and safety over racemates. However, S-form verapamil (S-Vera) reduces arterial pressure, has negative inotropic effects, increases left ventricular end-diastolic pressure and the regional chamber stiffness constant, and causes diastolic dysfunction [26]. Therefore, R-Vera is promising for DM treatment as its safety profile is well established and it induces fewer side effects than the racemate or S-Vera.

Nowadays, metformin (biguanide) and acarbose (α-glucosidase inhibitor) are widely used as first-line oral hypoglycemic agents (OHAs). Metformin lowers BG by decreasing intestinal glucose absorption and increasing peripheral glucose uptake. Acarbose reduces BG by competitively inhibiting α-glucosidases in the ileal brush border and α-amylase in the pancreas. Both metformin and acarbose can be administered alone or in combination with other OHAs or insulin. R-vera, as a stellar candidate for novel DM treatment, is designed to examine antidiabetic efficacy and compare it with monotherapy or add-on therapy of metformin/acarbose.

Here, we compared the cardiac effects of R-Vera and S-Vera by *in vitro* evaluating their calcium channel binding activity. R-Vera caused fewer CV effects than S-Vera. Proof-of-concept studies on hyperglycemia treatment was performed using T1DM and T2DM mouse models administered verapamil and R-Vera. We assessed the antidiabetic efficacy of verapamil and R-Vera and, moreover, in monotherapy and combination therapy. R-Vera plus metformin/acarbose co-administration had greater therapeutic efficacy than metformin/acarbose alone.

## Materials and methods

### *In vitro* study

Radioligand binding and tissue assays on R-Vera and S-Vera were designed in accordance with previous studies and were listed in Table 1. For atrial inotropy, ileal contractility and Somatostatin receptor type 2 (SSTR2) experiments, left atria and ilea were used and dissected from Dunkin Hartley guinea pigs (male or female, weighing 600 ± 80 g; National Laboratory Animal Center, Taiwan) based on previous studies [27–29]. For calcium channel L-type and sodium channel tests, the cerebral cortex or brain were dissected from Wistar rats (male, weighting 175 ± 25 g; BioLASCO Taiwan Co., Ltd., Taiwan). Detailed descriptions of the *in vitro* experiments are included in S1 Appendix.

**Table 1:** R-Vera and S-Vera test systems in *in vitro* radioligand binding and tissue assays.

Assay	Species/Tissue or Cell	Incubation buffer/Time	Reference
Calcium channel L-type, benzothiazepine	Wistar rat/Cerebral cortex	50 mM Tris-HCl (pH 7.4), 0.1% BSA/4°C for 3 h	[30]
Calcium channel L-type, dihydropyridine	Wistar rat/Cerebral cortex	50 mM Tris-HCl (pH 7.4)/25°C for 90 min	[31]
Calcium channel L-type, phenylalkylamine	Wistar rat/Brain	50 mM HEPES (pH 7.4)/25°C for 60 min	[32]
Calcium channel Ca_v_1.2	Human/HEK-293 cell	50 mM Tris-HCl (pH 7.4), 1 mM CaCl_2_, 0.1 mM PMSF/25°C for 2 h	[33, 34]
Sodium channel, site 2	Wistar rat/Brain	50 mM HEPES, 50 mM Tris-HCl (pH 7.4), 130 mM choline chloride, 5.4 mM KCl, 0.8 mM MgCl_2_, 5.5 mM Glucose, 40 μg/mL LqTX/37°C for 60 min	[35]
Calcium channel L-type, atrial inotropy	Guinea pig/Left atria	Tyrode, 0.6 mM calcium (pH 7.4)/32°C for 5 min	[27]
Calcium channel L-type, Ileum	Guinea pig/Ileum	Tyrode, 40 mM potassium depolarized, calcium-free (pH 7.4)/32°C for 5 min	[29]
Somatostatin receptor type 2 (SSTR2)	Guinea pig/Ileum	Krebs buffer (pH 7.4)/32°C for 5 min	[28]

### Animal studies

T1DM and T2DM mouse models were used to compare the relative efficacies of R-Vera and racemic verapamil. We used three experimental strategies to evaluate the antidiabetic effects of R-verapamil in type 1 and type 2 DM mouse models.

#### Experiment 1

**Fig 1:**
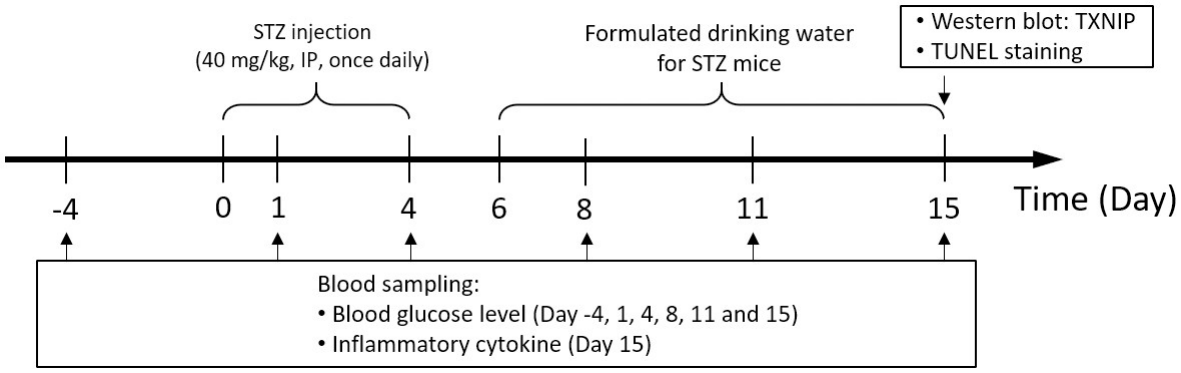
Scheme of STZ-induced T1DM mice administered racemic verapamil or R-Vera.

In Experiment 1 (T1DM model study; Fig 1), which was outsourced to and conducted by The Jackson Laboratory (Bar Harbor, Maine, US), male wild type C57BL/6J mice were fasted for 4 h before each STZ injection. All mice received intraperitoneal (i.p.) streptozotocin (STZ; 40 mg/kg in citrate buffer; pH 4.5; Sigma-Aldrich Corp., St. Louis, MO, U.S.A.) for 5 days [36]. R-Vera and racemic verapamil were dissolved into the drinking water for daily intake.

On study day 0, wild type C57BL/6J mice were administered low-dose (40 mg/kg) STZ using intraperitoneal injection for 5 days. All mice were randomly divided into four groups with eight mice per group. The Vehicle group comprised STZ-induced T1DM mice administered drinking water without verapamil or R-Vera. The V-100 group comprised STZ-induced T1DM mice administered 100 mg/mL/day verapamil. The RV-100 group comprised STZ-induced T1DM mice administered 100 mg/mL/day R-Vera. The RV-50 group comprised STZ-induced T1DM mice administered 50 mg/mL/day R-Vera for 10 days.

On study days 1, 4, 8, 11, and 15, mice were fasted for 6 h and their BG levels were measured using an AlphaTRAK 2 glucometer (Zoetis Inc., Madison, NJ, USA). Additional blood was collected and processed for serum isolation on day 15. The BG levels of Vehicle-treated STZ-mice on study days 8, 11, and 15 were measured to confirm the presence of hyperglycemia (> 300 mg/dL) in the T1DM mice model. The sera were stored at -80°C until IL-6 measurement with a SECTOR Imager 6000 (Meso Scale Diagnostics LLC, Rockville, MD, USA).

On study day 15, the mice were euthanized via CO_2_ asphyxiation and their pancreases were harvested for islet tissue isolation [37]. Western blot and transferase-mediated dUTP nick-end labeling (TUNEL) assays were performed to determine Txnip protein expression and β-cell apoptosis, respectively.

#### Experiment 2

**Fig 2:**
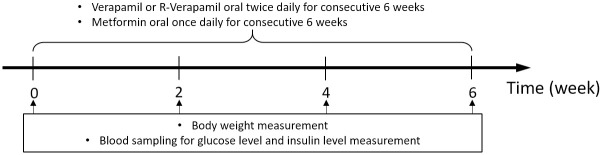
Scheme of *db*/*db* mice administered verapamil or R-Vera with or without metformin for 6 wks.

In Experiment 2 (T2DM model studies; Fig 2), which were outsourced to and conducted by Eurofins Pharmacology Discovery Services Taiwan, Ltd. (New Taipei City, Taiwan), nine- to ten-week-old male *db*/*db* mice (non-insulin-dependent diabetes mellitus, C57BLKS/J Iar- +Lepr^db^/+Lepr^db^) were acclimated for ≥ 1 week and grouped before treatment until the average BG was ≥ 350 mg/dL in animals fasted for 6 h. The *db/db* mice were orally gavage fed with R-verapamil combined with metformin, and BG and serum insulin levels were measured to evaluate the antidiabetic effect of R-verapamil in T2DM.

Six *db*/*m+* mice served as sham controls. All animals were provided access to drinking water *ad libitum* prior to the commencement of experiments. Groups, dosages, and administration frequencies are listed in Table 2. The mice were treated for 6 weeks.

**Table 2:** Dosages and dosing frequencies of drugs used in T2DM mouse model study.

Treatment strategy	Group	Route	Dosage	
			mg/kg/dose, frequency	mg/kg/day
N/A	Vehicle	PO	N/A, BID	N/A
Metformin-mono	Metformin	PO	300, QD	300
RV-mono	R-verapamil (RV-30)	PO	15, BID	30
	R-verapamil (RV-60)	PO	30, BID	60
	R-verapamil (RV-100)	PO	50, BID	100
	R-verapamil (RV-150)	PO	75, BID	150
V-mono	Verapamil (V-30)	PO	15, BID	30
	Verapamil (V-60)	PO	30, BID	60
RVM-combination	R-verapamil + Metformin (RV-30+Met)	PO	15, BID + 300, QD	30 + 300
	R-verapamil + Metformin (RV-60+Met)	PO	30, BID + 300, QD	60 + 300
	R-verapamil + Metformin (RV-100+Met)	PO	50, BID + 300, QD	100 + 300
Acarbose-mono	Acarbose-20	PO	20, QD	20
	Acarbose-40	PO	40, QD	40
RVA-combination	R-verapamil + Acarbose (RV-60+Aca-40)	PO	30, BID + 40, QD	60 + 40
VA-combination	Verapamil + Acarbose (V-60+Aca-40)	PO	30, BID + 40, QD	60 + 40

Acarbose-mono: *db*/*db* mice received acarbose only; BID: twice daily; Metformin-mono: *db/db* mice received metformin only; N/A: not applicable; PO: oral administration; QD: once daily; RV-mono: *db/db* mice received R-verapamil only; RVA-combination: db/db mice received R-verapamil and acarbose; RVM-combination: *db/db* mice received R-verapamil and metformin; V-mono: *db/db* mice received verapamil only; VA-combination: *db/db* received verapamil and acarbose.

Body weight, BG, and serum insulin were measured at weeks 0 (before treatment), 2, 4, and 6 in animals fasted for 6 h. All animals were euthanized via CO_2_ asphyxiation at experiment termination.

#### Experiment 3

**Fig 3:**
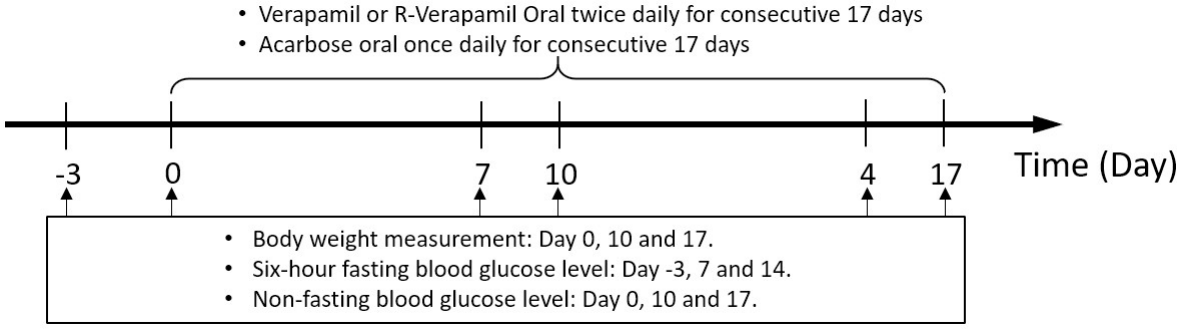
Scheme of *db*/*db* mice administered verapamil and R-Vera combined with acarbose.

To test the potential for combining R-vera with acarbose, we designed a pilot study in the form of Experiment 3. In Experiment 3 (T2DM model studies; Fig 3), which was outsourced to and conducted by Eurofins Pharmacology Discovery Services Taiwan, Ltd. (New Taipei City, Taiwan), nine- to ten-week-old male *db*/*db* mice (non-insulin-dependent diabetes mellitus, C57BLKS/J Iar- +Lepr^db^/+Lepr^db^) were acclimated for ≥ 1 week and grouped before treatment until the average BG was ≥ 350 mg/dL in animals fasted for 6 h. The *db/db* mice were orally gavage fed with R-verapamil combined with acarbose.

Six *db*/*m+* mice served as sham controls. Groups, dosages, and administration frequencies are listed in Table 2. Animals fasted for 6 h were administered the drugs for 17 days consecutively. Six-hour fasting BG was measured on days -3 (before treatment), 7, and 14. Non-fasting BG was measured on days 0 (before treatment), 10, and 17. Body weight was monitored thrice weekly during the study. All animals were euthanized via CO_2_ asphyxiation at experiment termination.

### Test drug preparation and administration route

The required amounts of R-Vera, S-Vera, and racemic verapamil hydrochloride were weighed and dissolved in sterilized ddH_2_O (Table 2). The drugs were prepared and administered as follows:

In the T1DM study (Experiment 1), the mice received verapamil or R-Vera via their drinking water. The formulated drinking water was freshly prepared daily during the study. The final R-Vera concentrations in the water were 0.5 mg/mL and 1 mg/mL, and the final racemic verapamil concentration in the drinking water was 1 mg/mL. The average doses were 50 mg/kg/day and 100 mg/kg/day R-Vera and 100 mg/kg/day verapamil, respectively [10].

In the T2DM study (Experiments 2 and 3), *db*/*db* mice were administered verapamil and R-Vera by oral gavage twice daily (*b*.*i*.*d*). Mono- and combination drug administration protocols were designed to represent various treatment strategies. In mono use, verapamil and R-Vera were administered to mice in distilled water twice daily (10 mL/kg of each dose). For combination use, various matches and dosages were evaluated for the RVM-combination (R-vera plus metformin in 1% Tween 80 administered once daily, both 10 mL/kg), the VA-combination (verapamil plus acarbose in 0.25% carboxymethylcellulose administered once daily, both 10 mL/kg), and the RVA-combination (R-vera plus acarbose in 0.25% carboxymethylcellulose administered once daily, both 10 mL/kg). The mono-use and combination-use dosages are shown in Table 2.

### Animals

Male wild type C57BL/6J mice (7 weeks; The Jackson Laboratory, Bar Harbor, ME, USA) were used in Experiment 1. Male C57BLKS/J Iar- +Lepr^db^/+Lepr^db^ mice [*db*/*db*; non-insulin-dependent diabetes mellitus (NIDDM); 50 ± 10 g, aged 9–10 weeks; Institute for Animal Reproduction (IAR), Ibaraki, Japan] were used in Experiments 2 and 3. The housing environment was maintained at a constant 22 ± 4°C and 55 ± 25% RH, respectively, under a 12-h light/dark cycle. The animals were maintained on a complete pellet diet and *ad libitum* tap water, and were acclimated for 1 week before the experiments. All animal study protocols were approved by the Institutional Animal Care and Use Committee (IACUC Protocol No. 19021-A1). All aspects of this work were conducted in general compliance with the Guide for the Care and Use of Laboratory Animals [38].

### Blood collection for BG, insulin, and cytokine measurements

Mice were either fasted for 6 h or fed, and their whole blood was collected from the tail tip or retro-orbital venous sinus. BG levels were measured using an AlphaTRAK 2 glucometer (Zoetis Inc., Madison, NJ, USA; T1DM mouse study) or an automated analyzer (TBA-120FR; (Toshiba Corp., Tokyo, Japan; T2DM mouse study). BG values were recorded in mg/dL.

For insulin and IL-6 measurements, 300 μL of whole blood was collected from the retro-orbital venous sinus of each mouse and placed in serum separator tubes (BD Microtainer; BD Bioscience, Franklin Lakes, NJ, USA) that were centrifuged at 2,000 ×*g* and 4°C for 5 minutes. The sera were transferred to 1.5-mL tubes and frozen at -80 °C until the next assay. The insulin and IL-6 levels were measured using ELISA [U-PLEX Biomarker Group 1 (Mouse) Multiplex Assay; MESO SCALE DISCOVERY^®^, Meso Scale Diagnostics LLC, Rockville, MD, USA] and SECTOR Imager 6000 (Meso Scale Diagnostics LLC, Rockville, MD, USA), respectively.

In Experiment 1, the IL-6 ratio to Vehicle of each group was calculated using the following formula: IL-6 ratio to Vehicle = (IL-6 concentration of group / IL-6 concentration of Vehicle) × 100%.

### Islet isolation

The isolation of pancreatic islets was performed as described by Carter et al. [37]. At the termination of the experiments, the pancreas was removed from CO_2_–euthanized STZ or *db/db* mice. The pancreas tissue was cut into 1–2 mm pieces and placed into 5 mL of digestive enzyme, which comprised 1.4 mg/mL Collagenase P (Roche, Indianapolis, IN, USA) in G-solution [Hank’s Balanced Salt Solution (HBSS; Invitrogen, Carlsbad, CA, USA), 0.35g/L NaHCO_3_, and 1% bovine serum albumin (BSA)]. The pancreatic pieces were enzymatically digested in the collagenase solution described above and they were concurrently mechanically digested by stirring in a 37°C water bath for 8–11 min. The digested lysate was quickly placed on ice and the lysate volume was made up to 15 mL with G-solution to dilute collagenase concentration and slow the digestive process. The mixture was centrifuged for 2 min at 290×g and the supernatant was discarded. This step was repeated twice. G-solution was then added to resuspend the islets to a concentration of 1 mg/mL.

### Western blotting

Islets were resuspended in 1 mg/mL of radioimmunoprecipitation assay (RIPA) buffer + protease inhibitor. Western blot was used to analyze TXNIP expression in the isolated islets. The reagents were mouse anti-TXNIP antibody (1:500; NBP1-54578; Novus Biologicals, Centennial CO, USA), mouse anti-β-actin (1:2,000; No. 3700; Cell Signaling Technology, Danvers, MA, USA), and a Wes^™^ Simple Western system (ProteinSimple, San Jose, CA, USA).

#### TUNEL immunostaining

One-quarter of each pancreas was fixed in formalin and embedded in paraffin for TUNEL immunohistochemical (IHC) staining [19, 39] using a Click-iT^™^ TUNEL colorimetric IHC detection kit (Invitrogen, Carlsbad, CA, USA) according to the product user guide. Apoptotic β-cells were counted using ImageJ 152a (NIH, Bethesda, MD, USA) [40].

### Data analysis and statistics

Multiple comparisons among body weight, BG, and serum insulin level were analyzed using one-way or two-way ANOVA followed by Tukey’s multiple comparison test and Student’s *t*-test in GraphPad Prism v. 8 (GraphPad Software, La Jolla, CA, USA). *P* < 0.05 was considered statistically significant. Data are expressed as means ± standard deviation (SD).

## Results

### Binding activity

Radioligand binding assay was performed to assess R-Vera and S-Vera binding activity to three different L-type calcium channels. The three calcium channels have different sensitivities to the same CCBs. Benzothiazepine (BZP), dihydropyridine (DHP), and phenylalkylamine (PLL) served as controls for the inhibition study and BZPcc, DHPcc, and PLLcc served as the L-type calcium channels. Numerous calcium channel binding responses indicated > 50% inhibition at the test concentration (Table 3). R-Vera more strongly inhibited BZPcc and DHPcc than S-Vera (90% vs. 66% and 85% vs. 66%, respectively). However, R-Vera and S-Vera showed similar PLLcc inhibition (104% vs. 99%).

**Table 3:** Radioligand assay of R-Vera and S-Vera binding on three L-type calcium channels.

Assay	R/S Form	Species	Concentration (μM)	Inhibition (%)
L-type calcium channel, benzothiazepine (BZPcc)	S	Rat	10	66
	R	Rat	10	90
L-type calcium channel, dihydropyridine (DHPcc)	S	Rat	10	66
	R	Rat	10	85
L-type calcium channel, phenylalkylamine (PLLcc)	S	Rat	10	99
	R	Rat	10	104

R-Vera had slightly stronger calcium channel inhibition than S-Vera. Statistically, however, R-Vera and S-Vera had similar calcium channel binding activity.

Ca_V_1.2 is a Ca_V_α1 subunit and a voltage-gated calcium channel of the L-type [41]. The IC_50_, inhibition constants (Ki), and Hill coefficients (nH) of the verapamil enantiomers were measured to evaluate the effects of blocking the Ca_V_1.2 channel on β-cell function. S-Vera showed slightly greater inhibition (56%) of the Ca_V_1.2 channel than R-Vera (47%) at the same concentration. IC_50_, K_i_, and nH were similar for both R-Vera and S-Vera (Table 4).

**Table 4:** Biochemical potency assessment of R-Vera and S-Vera on Ca_V_1.2 and sodium channel (Site 2). R-Vera and S-Vera had similar potency.

Biochemical Assay	R/S Form	Species	CONC. (μM)	INH. (%)	IC_50_ (μM)	Ki (μM)	nH
Calcium channel L-type, Ca_V_1.2	S	Human	300	56	228	108	1.34
	R	Human	300	47	> 300	N/A	N/A
Sodium channel (site 2)	S	Rat	10	88	2.22	2.02	1.21
	R	Rat	10	83	3.19	2.91	1.45

CONC.: concentration; INH.: inhibition; N/A: not available as values were outside test range.

Modulation of the calcium and sodium channels induces vasodilation, arrhythmia, angina, and electrolyte and water imbalances. IC_50_, K_i_, and nH were measured to assess the impact of verapamil enantiomers on the sodium channel (Site 2). IC_50_ and K_i_ were ~1.4× higher for R-Vera than S-Vera. The R-Vera nH was 1.2× higher than that of S-Vera according to the sodium channel assay (Table 4).

Tissue assays was conducted on atrial inotropy and ileal contractility to evaluate the potency of verapamil enantiomers on smooth muscle contraction via calcium channel and somatostatin receptor type 2 (SSTR2). R-Vera and S-Vera had similar inhibitory activity on various L-type calcium channels according to the radioligand binding assay. In the tissue studies, R-Vera was 9.0× and 3.4× weaker than S-Vera at inhibiting atrial inotropy and ileal contractility, respectively. R-Vera was 8.7× weaker than S-Vera as an agonist of SSTR2 and at inhibiting neurogenic ileal contraction (Table 4).

### *In vivo* studies on R-Vera in T1DM and T2DM mouse models

#### Experiment 1: RV-100 downregulated Txnip and preserved β-cells in STZ-induced mice (T1DM model)

**Fig 4:**
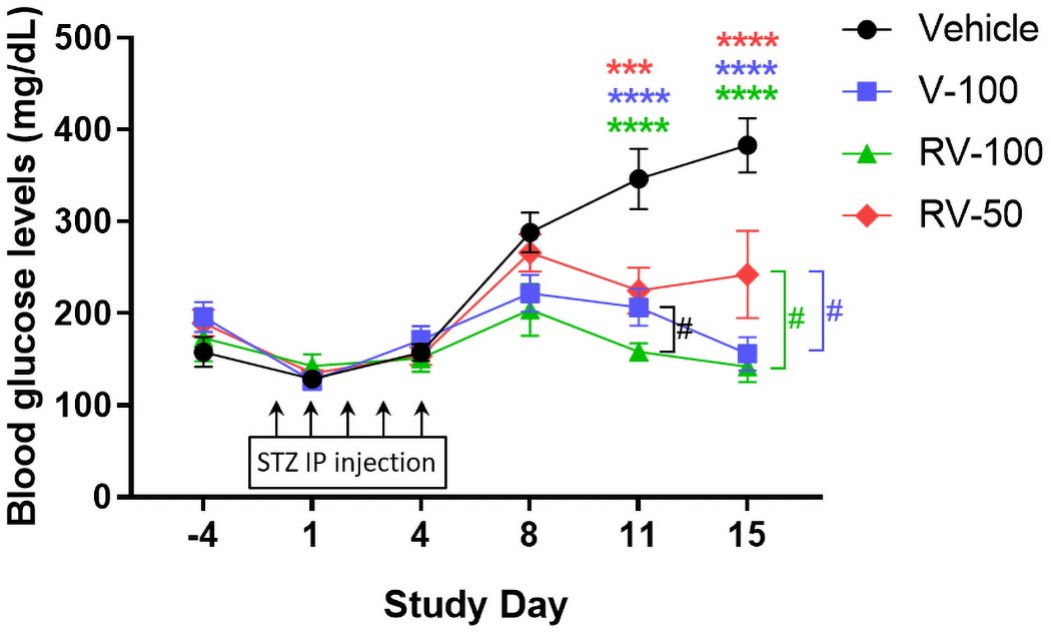
Fasting BG of STZ-induced mice treated with Verapamil (V) and R-Vera (RV) from days 6–15. Relative BG improved for the racemic and R-Vera groups. Bars are means ± SD. N = 8 mice per vehicle, V-100, RV-100, and RV-50 groups. *** *P*<0.001, ****P < 0.0001, significant difference from vehicle, using two-way ANOVA with Tukey’s multiple comparison; ^#^*P* < 0.05, significant difference between V-100 and RV-100 on day 11 and between V-100 or RV-100 and RV-50 on day 15, using an unpaired student’s *t*-test.

STZ-induced mice were administered drinking water containing racemic verapamil or R-Vera. Fasting BG levels were significantly reduced in V-100, RV-100, and RV-50 mice (compared to vehicle) by day 11 (vehicle vs. V-100, ****P < 0.0001; vehicle vs. RV-100, ****P < 0.0001; vehicle vs. RV-50, ***P < 0.001, Fig 4) and day 15 (vehicle vs. V-100, ****P < 0.0001; vehicle vs. RV-100, ****P < 0.0001; vehicle vs. RV-50, ****P < 0.0001; Fig 4). The fasting BG levels of RV-100 and V-100 also showed a marked reduction compared to RV-50 (V-100 vs. RV-50, ^#^*P* < 0.05; RV-100 vs. RV-50, ^#^*P* < 0.05; Fig 4). An unpaired *t*-test indicated that BG was significantly lowered by RV-100 compared to V-100 on day 11 (^#^*P* < 0.05; Fig 4). V-100, RV-100, and RV-50 treatment produced significantly lower BG levels than the vehicle group on days 11 and 15 (****P < 0.0001; Fig 4).

**Fig 5:**
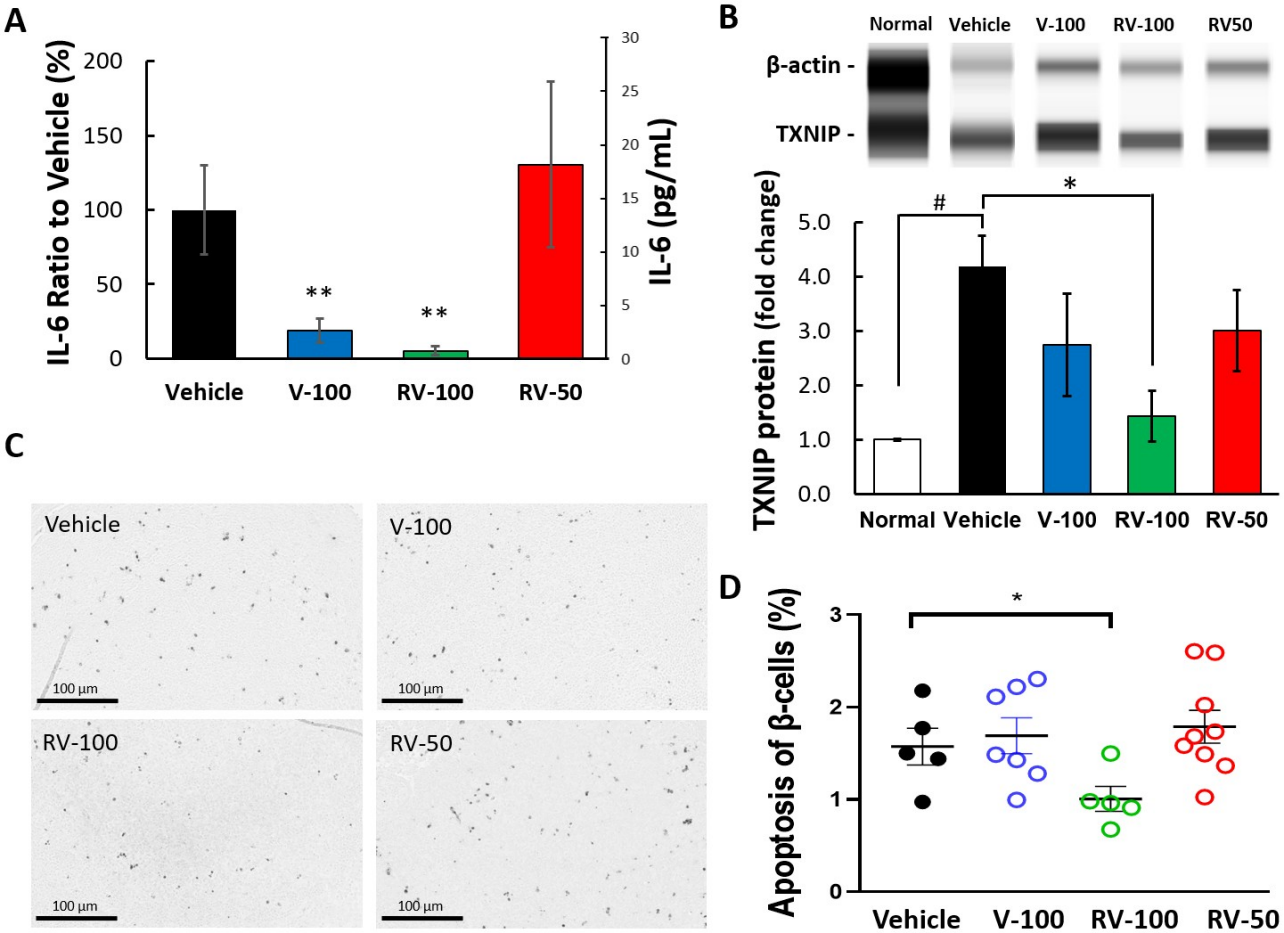
Proinflammatory cytokine (IL-6) and TXNIP expression and TUNEL assay for STZ-induced mice treated with Verapamil (V) and R-Vera (RV). **(A)** STZ-induced mice administered 100 mg/kg/day racemic or R-Vera exhibited significantly reduced serum IL-6 expression compared to vehicle mice whereas those administered 50 mg/kg/day R-Vera did not. **P < 0.01 indicates significant difference from vehicle using one-way ANOVA. N = 8 in each group. **(B)** STZ-induced mice showed highly upregulated islet tissue TXNIP compared to normal C57BL/6J mice. TXNIP was downregulated after racemic or R-Vera administration compared to vehicle. ^#^ *P* < 0.05 indicated significant difference between normal and vehicle; *P < 0.05 indicated significant difference to vehicle using one-way ANOVA. N = 8 in each group. **(C)** β-cell apoptosis assessed using TUNEL staining. Deep staining spots in images are apoptotic β-cells (200×). **(D)** TUNEL-positive cell counts show that STZ-induced mice administered 100 mg/kg/day R-Vera had significantly reduce β-cell apoptosis. *P < 0.05 indicated a significant difference between vehicle and RV-100 using one-way ANOVA. The sample numbers of 5, 7, 5, and 9 indicate the section numbers from the Vehicle, V-100, RV-100, and RV-50 groups, respectively (rather than the number of mice). Each data point represents the percentage of TUNEL–positive cells per slide in each group.

Mice administered multiple STZ doses showed significant relative increases in blood IL-6 levels [27]. Here, IL-6 expression was significantly reduced in the V-100 and RV-100 mice (**P < 0.01) compared to that in the vehicle control. However, IL-6 expression was similar for the RV-50 and vehicle mice (Fig 5A). The concentrations of IL-6 were 13.92 ± 4.28 (100%), 2.61 ± 1.12 (19%), 0.74 ± 0.38 (5%) and 18.17 ± 7.75 (131%) pg/mL in the Vehicle, V-100, RV-100, and RV-50 groups, respectively (the percentage was calculated as a normalized ratio to the Vehicle; Fig 5A). STZ-induced mice presented with progressive TXNIP upregulation and β-cell apoptosis [19]. In the present study, islet β-cell TXNIP expression was markedly higher in the vehicle mice than the normal C57BL/6J mice (^#^*P* < 0.05; Fig 5B). Mice presented with significant TXNIP downregulation in their islet tissues after 100 mg/kg/day R-Vera administration for 10 days (*P < 0.05; Fig 5B). The V-100 and RV-50 groups showed non-significant TXNIP downregulation in their islet tissues (Fig 5B). There were abundant and equal numbers of apoptotic (TUNEL-positive) cells in the Vehicle, V-100, and RV-50 mice. Nevertheless, apoptotic cells of the RV-100 mice were significantly less than that of Vehicle mice (*P < 0.05; Fig 5C and 5D). STZ-treated mice administered 100 mg/kg/day R-Vera displayed significantly reduced TXNIP expression and β-cell apoptosis in their islet tissues.

#### Experiment 2: RV-mono and RVM-combination improved BG in *db*/*db* mice

In Experiment 1, R-Vera improved BG and β-cell apoptosis in T1DM model mice. We also compared the efficacies of racemate and R-Vera in *db*/*db* mice (T2DM model). Here, *db*/*m+* mice served as sham control.

**Fig 6:**
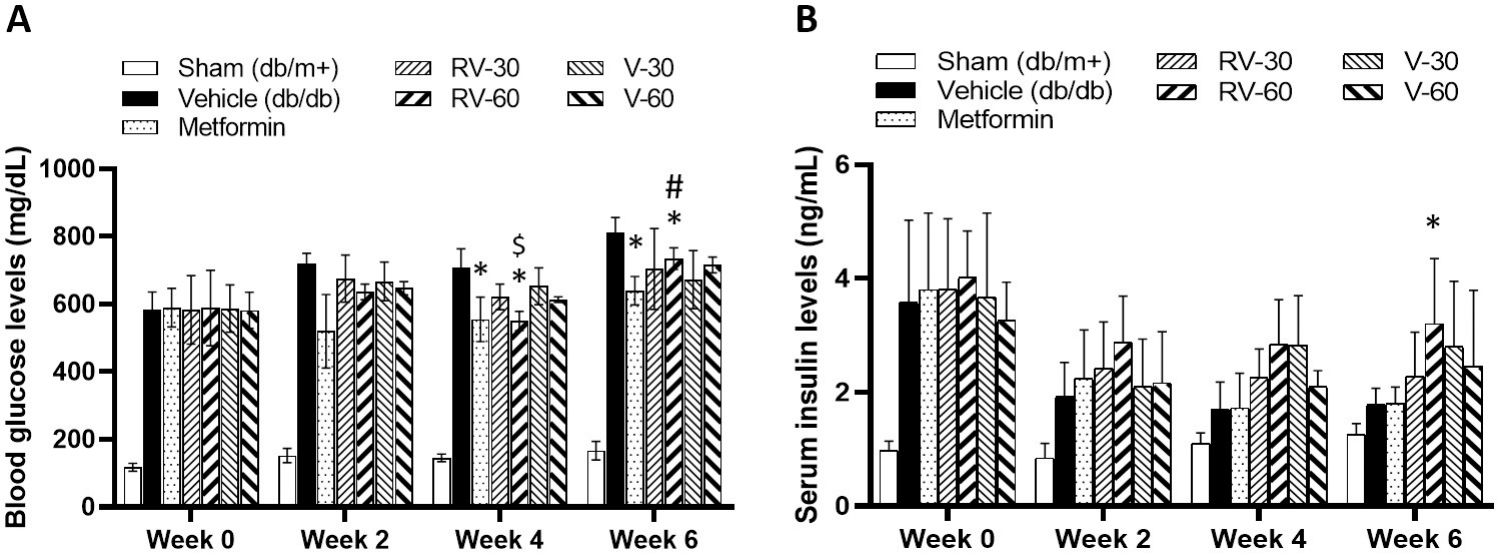
Effects of Verapamil (V) and R-Vera (RV) on BG and serum insulin in *db/db* mice. **(A)** BG and **(B)** serum insulin levels were measured on weeks 0 (before treatment), 2, and 4 and on day 6 in mice fasted 6 h. *P < 0.05 compared to vehicle (*db*/*db*); ^#^*P* < 0.05 compared to metformin; ^$^*P* < 0.05 compared to RV-30; two-way ANOVA. N = 5 per group except for n = 3 and n = 2 in V-60 group on weeks 2 and weeks 4 and 6, respectively, because of animal health issues. Data are expressed as means ± SD.

The Vehicle, Metformin, RV-30, RV-60, V-30, and V-60 mice all presented with similarly high BG at week 0. Vehicle mouse BG progressively increased on weeks 2, 4, and 6. Oral metformin administration significantly attenuated BG relative to the vehicle group (*P < 0.05; Fig 6A). Compared to the Vehicle, both verapamil and R-vera dosages produced similar BG levels by week 2. However, RV-60 significantly reduced BG relative to the Vehicle by week 4 (*P < 0.05). RV-60 significantly reduced BG to a greater extent than RV-30 by week 4 (^$^*P* < 0.05; Fig 6A). The BG levels in the RV-60 and V-60 mice were significantly lower than those in the vehicle mice by week 6 (*P < 0.05). Metformin was a positive control. The BG level in the metformin group was significantly lower than that of the vehicle by weeks 4 and 6. It is worth noting that, although RV-60 significantly reduced BG, metformin had a stronger effect at week 6 (^#^*P* < 0.05; Fig 6A).

The serum insulin levels in the vehicle and metformin mice declined to a level similar to that in the Sham mice by week 6 (Fig 6B). Verapamil and R-Vera administration generally maintained insulin levels more effectively than metformin. The serum insulin level in the RV-60 mice was significantly higher than that in the Vehicle mice at week 6 (*P < 0.05; Fig 6B).

The *in situ* hybridization results to assess *Txnip* mRNA expression are represented as a fold-change relative to expression in *db/m*^+^ mice, and are shown in S1 Fig. The fold-change in expression of *Txnip* mRNA in Vehicle, RV-30, RV-60, and V-60-treated *db/db* mice was 2.2, 2.0, 2.3 and 2.0, respectively, compared to that of vehicle-treated *db/m*^+^ mice. Although a slight decrease in *Txnip* mRNA expression was observed in all R-Vera-treated groups, no statistically significant difference in *Txnip* mRNA expression was observed in the islets of R-Vera or verapamil-treated *db/db* mice (*P* > 0.05; S1 Fig).

**Fig 7:**
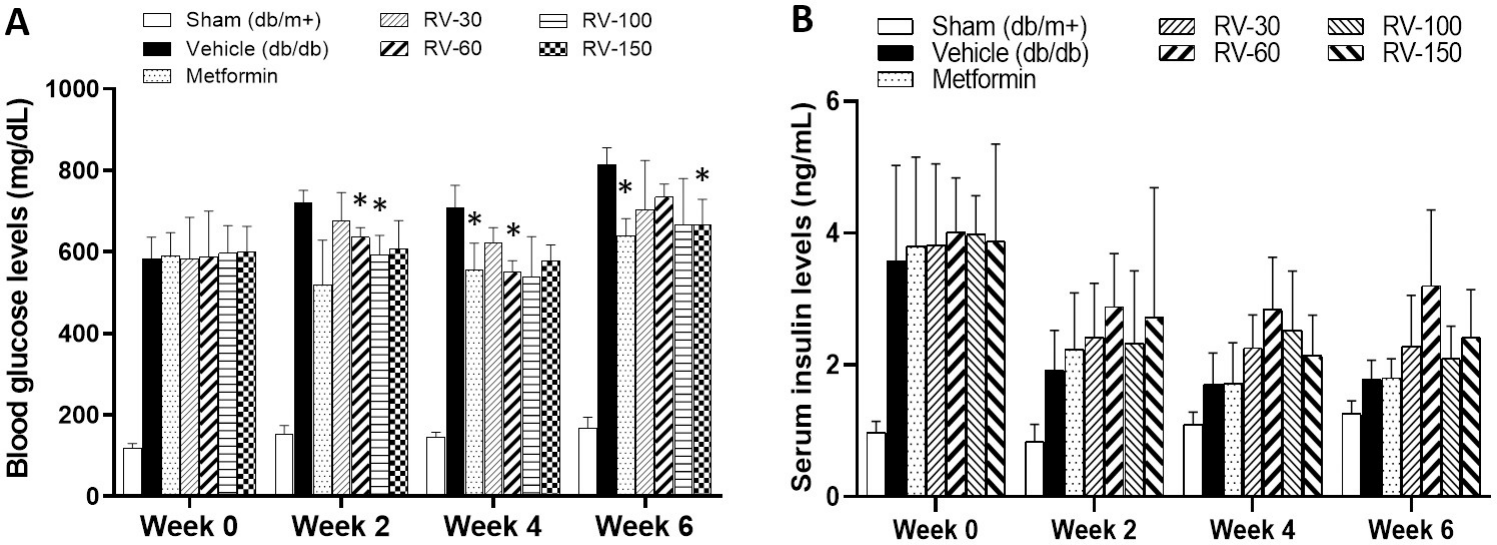
*db*/*db* mice treated with 30, 60, 100, and 150 mg/kg/day R-Vera (RV) for 7 weeks. *db*/*db* mice receiving metformin or RV showed (A) reductions in BG level and (B) slight improvements in serum insulin level. Nevertheless, no obvious dose-dependent effects on BG and serum insulin levels were found. N = 5 per group. *P < 0.05 compared to vehicle (*db/db*) group with two-way ANOVA.

In this study, we investigated R-Vera dose dependency. To this end, we administered 100 mg/kg/day and 150 mg/kg/day of the drug to *db*/*db* mice. The BG levels in the RV-60 and RV-100 mice were significantly lower than that in the vehicle mice at week 2 (*P < 0.05; Fig 7A). At week 4, the BG levels in the RV-100 and RV-150 mice decreased to a level similar to that in the RV-60 mice but only the RV-150 was significant (*P < 0.05; Fig 7A). At week 6, all R-Vera dosing levels improved BG to the same degree as that of metformin. However, only RV-150 mice presented with significant BG reduction compared to the Vehicle mice (*P < 0.05; Fig 7A).

The serum insulin level in the R-Vera mice was slightly higher than those in the vehicle and metformin mice but the differences were not statistically significant (Fig 7B).

We evaluated the glucose-lowering effect of RV-mono and RVM-combination on *db/db* mice. For RV-mono, the oral R-Vera doses were 15 mg/kg, 30 mg/kg, and 50 mg/kg twice daily which corresponded to 30 mg/kg/day, 60 mg/kg/day, and 100 mg/kg/day. For RVM-combination, the R-Vera doses were the same as those for RV-mono except 300 mg/kg metformin was co-administered once daily. Body weight, BG, and serum insulin were measured at week 0 (before treatment) and at weeks 2, 4, and 6 for animals fasted 6 h.

**Fig 8:**
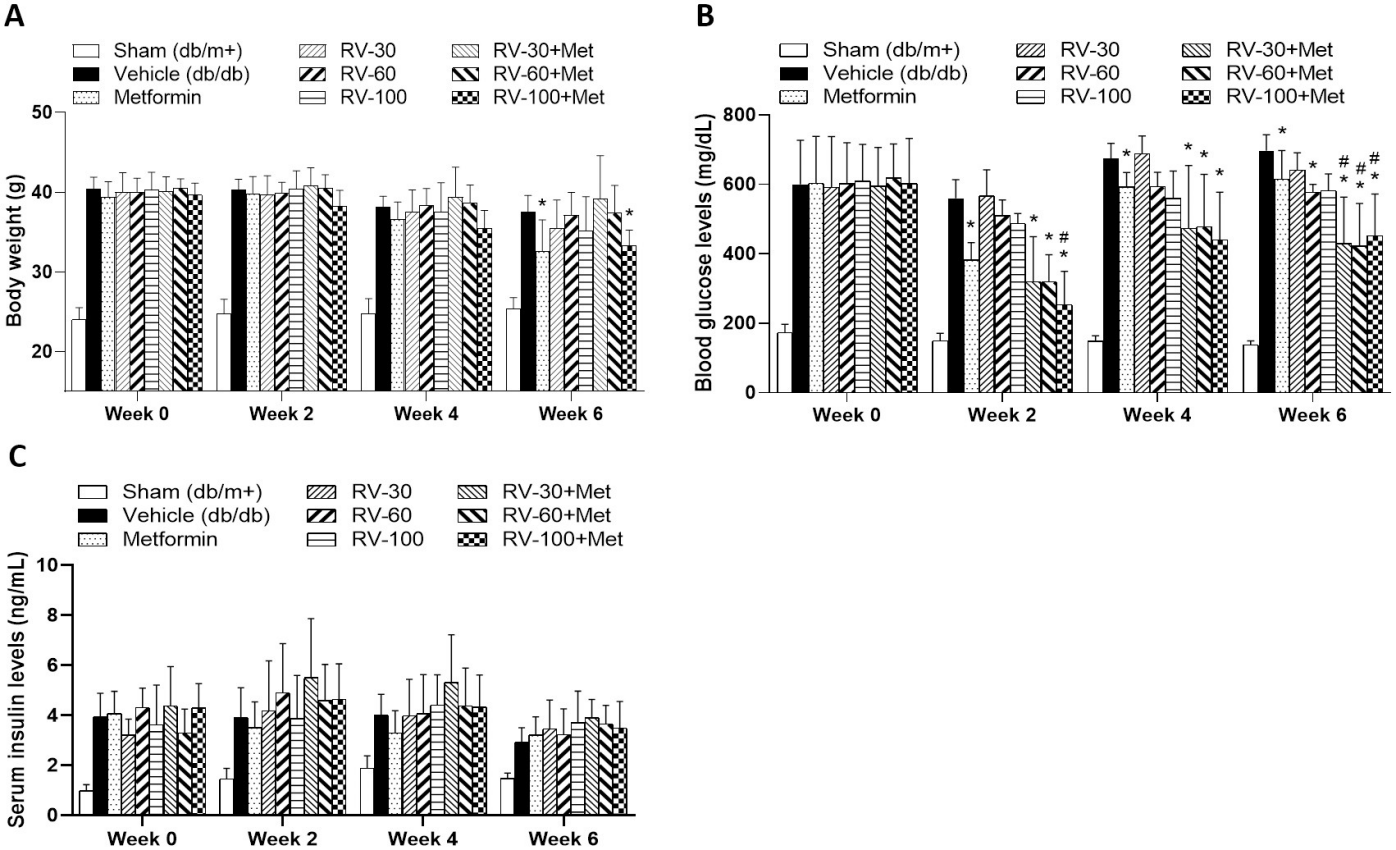
Effects of RV-mono and RVM-combination treatments in *db*/*db* mice. **(A)** The RV-100+Met and metformin groups displayed significant body weight reduction relative to the vehicle group (*P < 0.05; two-way ANOVA). **(B)** The RVM-combination treatment had significant glucose-lowering efficacy compared to the vehicle (*P < 0.05, two-way ANOVA) and the metformin treatment (^#^*P*<0.05, two-way ANOVA). **(C)** The serum insulin levels in the RVM-combination group were slightly higher than those in the metformin and RV-mono groups. N = 8 per group. Statistical analysis was performed using two-way ANOVA.

Metformin group body weight was significantly lower than that of the vehicle group at week 6 but did not differ from that of the sham group (*P < 0.05; Fig 8A). The RV-mono and RVM-combination groups presented with lower body weights than those of the vehicle group. However, only the RV-100+Met group displayed significantly lower body weight than the vehicle group (*P < 0.05) but it was similar to that of the metformin group (Fig 8A).

The RVM-combination and the metformin *db*/*db* mice exhibited significantly attenuated BG levels at weeks 2, 4, and 6 relative to the vehicle group (*P < 0.05; Fig 8B). The RV-mono *db*/*db* mice showed relatively reduced BG levels but only the measured BG reduction in the RV-60 group was significant at week 6 (*P < 0.05). Compared to the metformin *db*/*db* mice, those administered all RVM combination doses presented with significantly lower BG levels at week 6 (^#^*P* < 0.05; Fig 8B).

The serum insulin levels in the metformin-mono, RV-mono, and RVM-combination groups were similar at weeks 2, 4, and 6 (Fig 8C).

#### Experiment 3: RVA-combination improved *db*/*db* mouse body weight and BG

**Fig 9:**
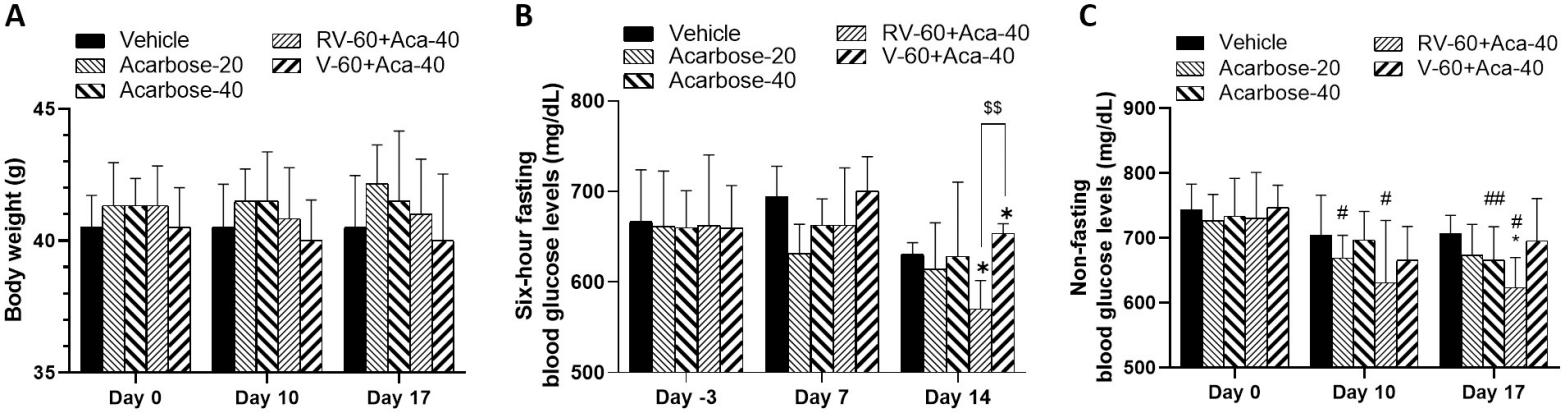
Effects of VA-combination, RVA-combination, and Acarbose-mono on *db*/*db* mouse body weight and BG. (A) Body weight changes (B) fasting BG levels (C) non-fasting BG levels. N = 6 in each group. *P < 0.05 indicates significant difference relative to vehicle; ^#^*P* < 0.05 and ^##^*P* < 0.01 indicate significant difference from day 0 in same group; ^$$^*P* < 0.01 indicates significant differences among groups.

By day 17, the *db*/*db* mice treated with the RVA-combination and VA-combination exhibited substantially lower body weights than those treated with Acarbose-mono. However, the values did not significantly differ among groups (Fig 9A). Compared to the Vehicle, the *db*/*db* mice treated with RVA-combination had significantly lower fasting BG levels on day 14 and non-fasting BG levels on day 17 (*P < 0.05; Fig 9B and 9C). Moreover, the fasting BG level of the RVA-combination group was significantly lower than that of the VA-combination group (^$$^*P* < 0.05; Fig 9B).

## Discussion

The Ca_V_1.2 channel on β-cells participates in insulin secretion and β-cell physiology and pathophysiology [41]. In our study, R-Vera was shown to have stronger inhibition than S-Vera on BZPcc and DHPcc (90% vs. 66% and 85% vs. 66%, respectively), but similar inhibition on PLLcc (104% vs. 99%). However, the difference of calcium channel inhibition is mild. R-Vera and S-Vera have similar binding activity on BZPcc, DHPcc, and PLLcc (Table 3), which indicates R-Vera and S-Vera might comparably inhibit TXNIP expression in the whole body.

Verapamil is a negative inotropic agent that decreases myocardial and peripheral smooth muscle contractility. We used a radioligand assay to examine the differences between verapamil enantiomers and distinguish their cardiac and neurogenic side effects. IC_50_, K_i_, and nH were evaluated to assess the influences of verapamil enantiomers on the sodium channel. Sodium channel is implicated in several CV reactions including vasodilation, arrhythmia, angina, and electrolyte and water balance. Here, R-Vera was 1.2× weaker than S-Vera at blocking sodium channels (Table 4). It is also reported that, the cardiac effect of verapamil is attributed mainly to S-Vera which is 8–20× stronger than R-Vera at slowing AV conduction and 15–50× stronger at reducing myocardial contractility in animals and humans [42]. Thus, R-Vera may have less CV effects than S-Vera.

In our radioligand assay, R-Vera was 8.7× weaker than S-Vera as an agonist of SSTR2 in the inhibition of ileal neurogenic contraction (Table 5). SSTR2 agonists inhibit neurogenically mediated contractions in isolated smooth muscle [43]. R-Vera showed a weaker inhibition of neurogenic smooth muscle contractions than S-Vera. Moreover, in our unpublished data of a toxicology study in dogs, the magnitude of PR interval prolongation was slightly greater in dogs treated with racemic verapamil than in those administered R-Vera. Unpublished data from our phase II clinical study investigating R-Vera as a T2DM treatment also disclosed that no hypotension or major adverse cardiovascular events (MACEs) occurred in patients treated with R-Vera for 3 months. For this reason, R-Vera appears to be a relatively safe option for T2DM patients.

**Table 5:** Tissue assay of R-Vera and S-Vera on L-type calcium channel and SSTR2. Agonistic and antagonistic activities were measured by isometric (atrial inotropy and SSTR2) and isotonic (ileal contractility) methods.

Assay	R/S form	CONC. (μM)	AG. (%)	ANT. (%)	IC_50_/EC_50_ (μM)
L-type calcium channel, atrial inotropy	S	10	0	78	3.7
	R	10	0	22	33.2
L-type calcium channel, ileal contractility	S	0.03	0	82	0.01
	R	0.03	0	44	0.034
Somatostatin receptor type 2 (SSTR2)	S	0.3	70	ND	0.15
	R	0.3	13	ND	1.30

CONC.: concentration; AG., agonist; ANT., antagonist; ND: assay not performed.

Verapamil may potentially treat DM. While calcium channel blockers (CCBs) were reported to reduce cardiac TXNIP expression, verapamil was also found to mediate TXNIP-lowering effects in pancreatic β-cells by decreasing *in vivo* binding of ChREBP, which acts as a key transcription factor controlling β-cell TXNIP transcription [19]. In our study, 10 μM R-Vera was shown to inhibit BZPcc, DHPcc, and PLLcc by 90%, 85% and 104%, respectively. Verapamil doses of 50 μM, 100 μM, and 150 μM were shown to significantly inhibit TXNIP mRNA expression in human islets, and Xu et al. demonstrated that this downregulation of TXNIP expression resulted from decreased intracellular calcium levels [19]. According to our study (Tables 3 and 4), R-Vera and S-Vera exhibit a similar capacity to inhibit calcium channels. We hence infer that R-Vera partially inhibits TXNIP expression and that 100 mg/mL/day of R-Vera may have antidiabetic effects similar to the results reported by Xu et al. [19]. In the present study, R-Vera markedly downregulated Txnip and reduced β-cell apoptosis in T1DM mice (Fig 5). Mice administered with 100 mg/kg/day R-Vera for 10 days demonstrated significant Txnip downregulation in their islet tissues (*P < 0.05; Fig 5B). Coherently, there were significantly fewer apoptotic cells in the RV-100 mice than the vehicle mice (*P < 0.05; Fig 5C and 5D). These results support those by Xu et al. that indicates the potential of R-vera to preserve β-cells [19].

In the T1DM study (Experiment 1), 100 mg/kg/day R-vera and racemic verapamil had similar efficacy in terms of lowering BG and IL-6 in STZ-induced mice. However, 50 mg/kg/day R-Vera had less efficacy (Figs 4 and 5A). The 100 mg/kg/day R-Vera dosage significantly downregulated Txnip, based on the results from the western blot, and prevented β-cell apoptosis based on the results from the IHC assay (Fig 2B–2D).

TXNIP is known as a major regulator of the cellular redox state. Several studies have reported that TXNIP overexpression induces apoptosis in pancreatic β-cells [17, 44] and TXNIP-mediated cardiomyocyte apoptosis is highly involved in the pathogenesis of diabetic cardiomyopathy [45]. Chen et al. also showed that the administration of racemic verapamil reduces cardiac expression of Txnip and cleaved caspase-3 in the hearts of STZ-induced diabetic mice. They also reported that the cardiomyocytes of verapamil-treated mice exhibited significantly fewer TUNEL–positive nuclei compared to the STZ control. Based on these data, we employed a TUNEL assay to investigate the inhibition of Txnip-induced β-cell apoptosis in our study. Since our results showed that R-Vera inhibits Txnip expression to a degree that is similar to racemic verapamil, R-Vera may also reduce the expression of cleaved caspase-3 in our STZ-treated mice model. R-Vera may act like verapamil, which downregulates TXNIP by binding the TXNIP promoter [19] and ameliorating TXNIP-induced mitochondrial β-cell apoptosis [39].

Calcium channels on β-cells play the same role despite the DM types, suggesting that R-Vera may ameliorate β-cell apoptosis in the T2DM mouse model as well. Although the disease progression is different in T1DM compared to T2DM, which also apparent in the results from different animal models, the antidiabetic effects of inhibiting TXNIP can be investigated by measuring insulin levels. The insulin and C-peptide levels in *db*/*db* mice tend to rise around 10-weeks-old due to insulin resistance, yet this is followed by a reduction that occurs concurrently with an initial decrease in β-cell mass during weeks 14 to 18 [46]. Despite the predictable reduction in insulin and C-peptide levels during weeks 14 to 18 in *db*/*db* mice, serum insulin levels were higher in *db*/*db* mice administered R-Vera (Experiment 2, the T2DM model) than they were in mice administered metformin and vehicle, and moreover, these levels were maintained through weeks 4 to 6 (Figs 6B and 7B). Although the changes in insulin levels were not statistically significant, there was an observable trend in the *db*/*db* mice experiment, which suggests that R-vera may help to preserve endogenous insulin levels during the development of T2DM.

Due to the nature of the metabolic mediators which produce responses in tissues, there are both metabolite and process variations in the measurement of insulin, and it is worth noting that this parameter has limits for the evaluation of antidiabetic effects in T2DM. In addition, alternative mechanisms are being explored to investigate the ability of verapamil to reduce BG. Verapamil is known as a calcium channel blocker which improves insulin-stimulated glucose transport in skeletal muscle of the obese Zucker rat, proving its effects on glycemic control and insulin sensitivity [47]. R-Vera may effectively prevent β-cell loss, nevertheless, our investigations were limited by the lack of a suitable T2DM animal model in which to examine TXNIP expression and β-cell apoptosis.

In the T2DM study (Experiment 2), R-Vera lowered BG in *db*/*db* mice in the dose range of 30–100 mg/kg/day (Figs 7 and 8). However, there does not seem to be a dose-response relationship, as 60 mg/kg/day R-Vera had the greatest effect on BG level and insulin level (without significance but a trend between dosage groups) and was hence selected as the optimal dosage to test in Experiment 3.

In light of the finding that R-vera increases insulin level in T2DM mice, adjunct therapy of R-vera was investigated. The theoretical aims of administering R-Vera/racemic verapamil as an adjunct therapy for metformin/acarbose are to enhance glucose reduction relative to that which is provided by metformin/acarbose alone and to protect β-cells from TXNIP-induced apoptosis. Here, the R-Vera or racemic verapamil combination strategy significantly lowered BG (Figs 8 and 9) and the RVA-combination demonstrated better efficacy than the VA-combination. Notably, Moreover, the labels of metformin (Glucophage SR) and acarbose (PRECOSE^®^) contraindicated their combination with verapamil. Metformin labeling and recent studies showed that verapamil may inhibit organic cation transporters (OCT) 1 and 2, reduce metformin efficacy, and decrease OCT2-mediated renal metformin elimination [48, 49].

In one case report, clinical hypoglycemia treatment in bariatric surgery patients was initiated with acarbose and verapamil. Hence, acarbose and verapamil co-administration may raise BG as suggested on the label [50]. To test the potential of combining R-Vera with acarbose, we designed a pilot study in the form of Experiment 3. In the present study, BG in the *db/db* mice administered the VA-combination was higher than that in the *db/db* mice administered Acarbose-mono. Nevertheless, *db/db* mice administered with the RVA-combination displayed markedly reduced BG compared to those administered Acarbose-mono (Fig 9). The complete mechanism of action of R-Vera remains to be elucidated. However, our ongoing findings indicate that R-Vera provides evidence-based benefit in the improvement of BG levels. Co-administration with R-vera has better antidiabetic effects.

R-vera may have potential to treat inflammation. Systemic inflammation is often associated with obesity/insulin resistance, T1DM, and T2DM [51–53]. DM animal models such as STZ-induced and *db*/*db* mice presented with upregulated proinflammatory cytokines such as TNF‑α, IL-6, and IL-1β compared to normal mice [36, 54]. Diabetes-induced myocardial injury was highly correlated with serum and myocardial proinflammatory cytokines [54]. In Experiment 1, we assessed serum IL-6 in STZ-induced mice. Verapamil and R-Vera (100 mg/kg/day) significantly downregulated IL-6 whereas 50 mg/kg/day R-Vera did not (Fig 5A). Therefore, R-Vera and verapamil have potential to protect against diabetic myocardial injury and ameliorate islet inflammation.

Cardiovascular agents must be carefully administered to patients with DM, since DM might increase the relative risk of succedent cardiac death, which is the most common cause of death in patients with DM (N = 118/669; 18%) [55]. Nevertheless, these drugs may be vital for T2DM patients with CV comorbidities such as hypertension and atherosclerosis. Hypertension is a strong risk factor in the development of CV disease and remains a leading cause of morbidity and mortality in patients with T1DM and T2DM [56, 57]. Patients with diabetes benefit from blood pressure control when they have a high absolute CV risk. This recommendation by the American Diabetes Association is consistent with guidelines from the American College of Cardiology/American Heart Association [56].

The study by Rosenthal et al. showed that verapamil significantly reduces systolic blood pressure from 175 ± 9 mmHg to 149 ± 19 mmHg in diabetic hypertensive rats [58]. In addition, verapamil was also found to markedly inhibit increases in heart rate and blood pressure (including diastolic pressure, systolic pressure, and mean blood pressure) in high-fat diet-fed mice [59]. In our unpublished data from a toxicology study in dogs, R-Vera mildly slowed the heart rate and decreased systolic blood pressure, diastolic blood pressure, and mean blood pressure. The antihypertensive effects of R-Vera resembled those of verapamil. In view of the latent side effects associated with S-Vera as previously discussed, R-vera has CV-benefits rather than CV-side effects and is a comparatively better enantiomer to use in the development of a new DM drug.

Oral R-Vera might be a safe and effective novel approach for the treatment of DM. The present and earlier studies indicated that R-Vera shows promising efficacy at improving glycemic control, preserving endogenous β-cell function, and reducing insulin dependency. R-Vera is a relatively safe option for DM and other CV comorbidities compared to its racemate and S-Vera. Thus, R-Vera merits further clinical investigation as a next-generation DM treatment that could delay or arrest disease progression and improve patient quality of life.

## Supporting information

S1 Appendix Supplementary materials and methods for *in vitro* studies. (DOCX)

S1 Fig Effects of Verapamil (V) and R-Vera (RV) on *Txnip* mRNA expression in *db/db* mice. Quantification of *Txnip* mRNA expression in islets from pancreatic tissue. *Txnip* mRNA levels in the db/m+ Vehicle group were significantly lower than that of the other groups (*** *P* < 0.001). No statistical difference in *Txnip* mRNA expression was observed between the *db/db* Vehicle, RV-30, RV-60, and V-60 groups (n = 8 in each group; one-way ANOVA with Tukey’s multiple comparison). Data are expressed as means ± SD.
(TIF)
