# Supplementary material for: Evaluating the antidiabetic effects of R-verapamil in type 1 and type 2 diabetes mellitus mouse models
Source: PLoS One. 2021 Aug 6;16(8):e0255405. doi: 10.1371/journal.pone.0255405 (PMC8345870; doi:10.1371/journal.pone.0255405)
Supplement: S1 Appendix — (DOCX) [file pone.0255405.s001.docx]

**S1 Appendix**

**Supplementary materials and methods for *in vitro* studies**

The *in vitro* studies were conducted by Eurofins Panlabs Discovery Services Taiwan, Ltd. (New Taipei City, Taiwan). The following brief methods were provided by Eurofins.

**Calcium channel L-type, atrial inotropy [Test Number: 415000] [18]**

Left atria obtained from Dunkin-Hartley–derived male or female guinea pigs (National Laboratory Animal Center, Taiwan) weighing 600 ± 80 g and sacrificed by CO_2_ overexposure were used for these tests. The tissue was removed and placed under 1 g tension in a 10 mL bath containing Tyrode’s solution pH 7.4 with one-third normal (0.6 mM) calcium at 32°C and subjected to field stimulation (70% of maximum voltage, 1.5 Hz, 0.2 msec). An R- or S-verapamil (30 µM)-induced isometrically recorded positive inotropic response by 50 percent or more (≥50%) within 5 min (relative to the response to the 0.6 mM CaCl_2_ control) indicated possible calcium L channel receptor agonist activity.

At a test concentration of R- or S-verapamil where no significant agonist activity was seen, the ability to inhibit the 0.6 mM CaCl_2_ -induced positive inotropic response by 50 percent or more (≥50%) indicated calcium L channel receptor antagonist activity.

**Calcium Channel L-Type, Ileum [Test Number: 415510] [20]**

A segment of ileum obtained from Dunkin-Hartley–derived male or female guinea pigs weighing 600 ± 80 g (National Laboratory Animal Center, Taiwan) and sacrificed by CO_2_ overexposure was used for this test. The tissue was placed under 1 g tension in a 10 mL bath containing calcium free, potassium (40 mM) depolarized modified Tyrode solution pH 7.4 at 32°C. An R- or S-verapamil (30 µM)-induced isotonically recorded contraction by 50 percent or more (≥50%) within 5 min, relative to the control 0.6 mM CaCl_2_ response, indicated possible calcium L channel receptor agonist activity.

At a test concentration of R- or S-verapamil where no significant agonist activity was seen, the ability to reduce the 0.6 mM CaCl_2_ -induced contractile response by 50 percent or more (≥50%) indicated calcium L channel receptor antagonist activity.

**Calcium channel L-type, benzothiazepine [Test Number: 214510] [21]**

Brains (except cerebella) of male Wistar–derived rats weighing 175 ± 25 g (BioLASCO Taiwan Co., Ltd., Taiwan) were used to prepare L-type benzothiazepine calcium channels in modified Tris-HCl buffer pH 7.4. A 0.5 mg aliquot was incubated with 2 nM [^3^H]Diltiazem for 180 minutes at 4°C. Non-specific binding was estimated in the presence of 10 µM diltiazem. Membranes were filtered and washed, and the filters counted to determine specific binding of [^3^H]Diltiazem. Compounds were prepared at 10 µM concentrations for screening.

**Calcium channel L-type, dihydropyridine [Test Number: 214600] [22]**

Cerebral cortices of male Wistar–derived rats weighing 175 ± 25 g (BioLASCO Taiwan Co., Ltd., Taiwan) were used to prepare L-type dihydropyridine calcium channels in Tris-HCl buffer pH 7.4. A 2.5 mg aliquot was incubated with 0.1 nM [^3^H]Nitrendipine for 90 minutes at 25°C. Non-specific binding was estimated in the presence of 1 µM nitrendipine. Membranes were filtered and washed, and the filters were counted to determine specific binding of [^3^H]Nitrendipine. Compounds were prepared at 10 µM concentrations for screening.

**Calcium channel L-type, phenylalkylamine [Test Number: 215000] [23]**

Whole brain membranes of male Wistar–derived rats weighing 175 ± 25 g (BioLASCO Taiwan Co., Ltd., Taiwan) were used to prepare L-type phenylalkylamine calcium channels in HEPES buffer pH 7.4. A 10 mg aliquot was incubated with 0.4 nM [^3^H](-)-Desmethoxyverapamil (D-888) for 60 minutes at 25°C. Non-specific binding was estimated in the presence of 10 µM D-600. Membranes were filtered and washed, and the filters were counted to determine specific binding of [^3^H](-)-Desmethoxyverapamil (D-888). Compounds were prepared at 10 µM concentrations for screening.

**Calcium channel Cav1.2 [Test Number: 214320] [24, 25]**

Human recombinant voltage gated calcium channel Cav1.2 receptors expressed in HEK293 cells were prepared in modified Tris-HCL buffer pH 7.4. A 40 μg aliquot was incubated with 0.7 nM [^3^H]PN200-110 for 120 minutes at 25°C. Non-specific binding was estimated in the presence of 1 µM Isradipine. Receptors were filtered and washed, and the filters were counted to determine specific binding of [^3^H]PN200-110. Compounds were prepared at 10 µM concentrations for screening.

**Sodium channel, site 2 [Test Number: 279510] [26]**

Whole brains (except cerebellum) of male Wistar–derived rats weighing 175 ± 25 g (BioLASCO Taiwan Co., Ltd., Taiwan) were used to prepare site 2 sodium channels in modified HEPES/Tris-HCl buffer pH 7.4 containing 40 μg/ml LqTx (alpha scorpion toxin from *Leiurus quinquestriatus*). A 7.5 mg aliquot was incubated with 5 nM [^3^H]Batrachotoxinin for 60 minutes at 37°C. Non-specific binding was estimated in the presence of 100 μM veratridine. Membranes were filtered and washed, and the filters counted to determine specific binding of [^3^H]Batrachotoxinin. Compounds were prepared 10 μM concentrations for screening.

**Somatostatin receptor type 2 (SSTR2) [Test Number: 476200] [19]**

A segment of ileum obtained from Dunkin-Hartley–derived male or female guinea pigs weighing 600 ± 80 g and sacrificed by CO_2_ overexposure was used for this test. The tissue was placed under 0.5 g tension in a 10 mL bath containing Krebs solution pH 7.4 at 32°C and subjected to field stimulation (70% of maximum voltage, 0.1 Hz, 0.3 msec). An R- or S-verapamil (30 µM)-induced reduction of isometrically recorded contractions by 50 percent or more (≥50%) within 5 min, relative to the control 0.03 µM somatostatin 28 response, indicated possible somatostatin sst_2_ receptor agonist activity.

At a concentration of R- or S-verapamil where no significant agonist activity was seen, the ability to inhibit somatostatin_28_ -induced relaxant response by more than 50% indicated somatostatin sst_2_ receptor antagonist activity.

***Txnip* mRNA *in situ* hybridization in *db/db* mice**

As part of our T2DM investigations, we examined the expression of *Txnip* using *in situ* hybridization. After *db/db* mice were sacrificed, the collected pancreas samples were immersion-fixed in 10% neutral buffered formalin containing 4% formaldehyde. After 24 hours of fixation, pancreas sections were carefully dissected from the formalin-fixed block sample. Two sections were cut from each block, sampled 300 µm apart, and each section was arranged on one microscope slide, representing in total a systematic, uniform random sampling of the available pancreas tissue.

An RNAscope 2.5 HD - RED Assay (Advanced Cell Diagnostics, Inc., Newark, CA, USA) was used to visualize *Txnip* mRNA in the pancreas tissue. Slides with pancreas tissue were processed according to the RNAscope 2.5 HD – RED Assay user manual. After *in situ* hybridization, the slides were counterstained in Gill's hematoxylin and sealed with a coverslip. The slides were scanned under a 20× objective in a ScanScope AT slide scanner (Aperio, Leica Biosystems Nussloch Gmb).

*Txnip* mRNA expression in the exocrine and endocrine pancreas was quantitively evaluated using Visiomorph pathology image analysis software (Visiopharm, Denmark). Visiomorph protocols are designed to detect labelled mRNA and quantitative estimates are expressed as mRNA densities (mRNA per unit area of tissue). The comparison of *Txnip* expression between the experimental groups was expressed as a fold-change value relative to the Vehicle control.
