# Supplementary figures and images for: Evaluating the antidiabetic effects of R-verapamil in type 1 and type 2 diabetes mellitus mouse models
Source: PLoS One. 2021 Aug 6;16(8):e0255405. doi: 10.1371/journal.pone.0255405 (PMC8345870; doi:10.1371/journal.pone.0255405)

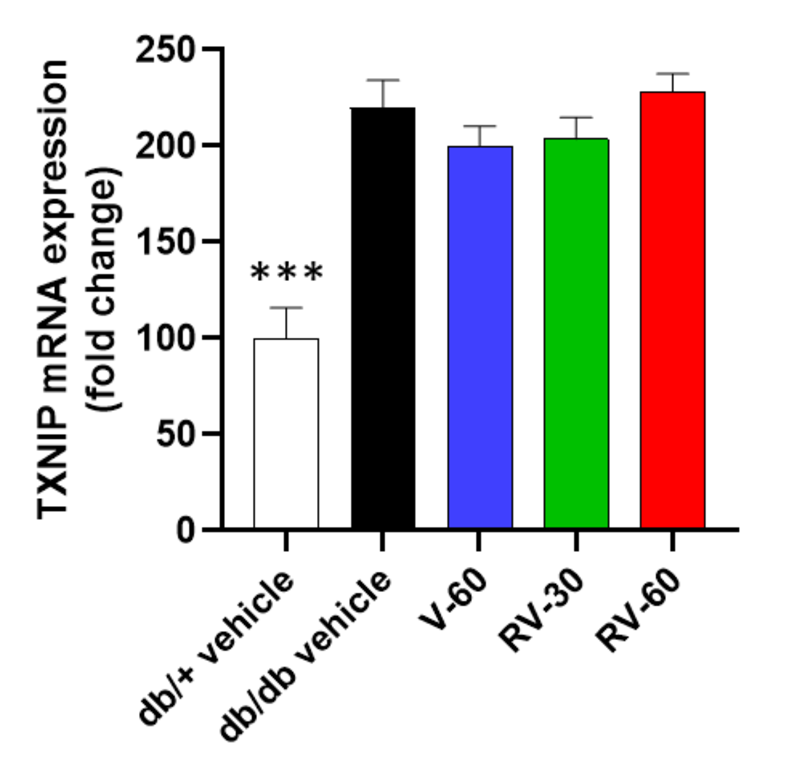

Supplement: S1 Fig — Quantification of Txnip mRNA expression in islets from pancreatic tissue. Txnip mRNA levels in the db/m+ Vehicle group were significantly lower than that of the other groups (*** P < 0.001). No statistical difference in Txnip mRNA expression was observed between the db/db Vehicle, RV-30, RV-60, and V-60 groups (n = 8 in each group; one-way ANOVA with Tukey’s multiple comparison). Data are expressed as means ± SD. (TIF) [file pone.0255405.s002.tif]
